# Nitrogen Uptake by Two Plants in Response to Plant Competition as Regulated by Neighbor Density

**DOI:** 10.3389/fpls.2020.584370

**Published:** 2020-12-10

**Authors:** Xuan Jia, Chaohe Huangfu, Dafeng Hui

**Affiliations:** ^1^Anhui Province Key Laboratory of Wetland Ecosystem Protection and Restoration, School of Resources and Environmental Engineering, Anhui University, Hefei, China; ^2^Department of Biological Sciences, Tennessee State University, Nashville, TN, United States

**Keywords:** nitrogen uptake, organic nitrogen, plant competition, neighbor density, functional traits

## Abstract

Plant species may acquire different forms of nitrogen (N) to reduce competition for the same resource, but how plants respond to neighbors with different densities in their N uptake is still poorly understood. We investigated the effects of competition regime on the uptake of different N forms by two hygrophytes, *Carex thunbergii* and *Polygonum criopolitanum*, by conducting a hydroponic test of excised roots and an *in situ* experiment in a subtropical wetland ecosystem. The two species were grown either in monocultures or mixtures with various neighbor densities. Root functional traits and N uptake rates of different N forms were measured. Our results showed that N uptake was mainly determined by N form, rather than species identity. Both species were able to use organic N sources, but they took up relatively more N supplied as NO${}_{3}^{-}$ than as NH${}_{4}^{+}$ or glycine, irrespective of competition treatments. Both species preferred NO${}_{3}^{-}$ when grown in monoculture, but in the presence of competitors, the preference of fast-growing *C. thunbergii* persisted while *P. criopolitanum* acquired more NH${}_{4}^{+}$ and glycine, with stronger responses being observed at the highest neighbor density. The hydroponic test suggested that these divergences in N acquisition between two species might be partially explained by different root functional traits. To be specific, N uptake rates were significantly positively correlated with root N concentration and specific root length, but negatively correlated with root dry matter content. Our results implicated that *C. thunbergii* has a competitive advantage with relatively more stable N acquisition strategy despite a lower N recovery than *P. criopolitanum*, whereas *P. criopolitanum* could avoid competition with *C. thunbergii* via a better access to organic N sources, partly mediated by competition regimes.

## Introduction

Nitrogen (N) is an essential element for plant growth, and its availability limits the productivity of most terrestrial ecosystems (Lebauer and Treseder, 2008). N limitation is common, mainly because the inorganic N (NO${}_{3}^{-}$ and NH${}_{4}^{+}$) contents in the soil are low and a large fraction of soil N exists in organic form which is not readily accessible to plants (Jiang et al., 2017). In the past 30 years, an increasing number of studies have indicated that it might be a common phenomenon for plants to take up low molecular-weight organic N directly from soil solution (Näsholm et al., 2009). Plants may use distinct nutrient acquisition strategies in response to changing environment, which is closely correlated with species co-existence (McKane et al., 2002) and ecosystem functioning (Boudsocq et al., 2012). Such strategies include that of nutrient uptake, transportation, utilization, and resorption. Due to the differences in mobility, energy consumption in assimilation, characteristics of various N transport genes of different N forms, and tolerance to ion toxicity among species (Zhang et al., 2018), plant species have different capacities in taking up NO${}_{3}^{-}$, NH${}_{4}^{+}$, or organic N as their primary N source. Some species have a preference for specific N forms (McKane et al., 2002), while other species showed flexibility in N uptake (Houlton et al., 2007). Whether a plant can change its N acquisition pattern will largely determine its ability to adapt to environmental changes (Gao et al., 2020). Studies on the acquisition of organic N by plants are mainly focused on alpine and arctic ecosystems with slow N mineralization rate (e.g., McKane et al., 2002; Xu et al., 2006). Recently, studies have confirmed that tropical forest species can also take up organic N (Andersen et al., 2017; Liu et al., 2018). Given that the N acquisition pattern may be ecosystem specific (Zhou et al., 2019), it is not clear whether the differences in N acquisition strategies among coexisting plants apply across different ecosystems (Yang et al., 2018).

The complexity of N acquisition strategies largely stems from the interplay between plant species and their interactions with various abiotic variables (Moreau et al., 2019). On the one hand, all species in the community are facing resource competition from other coexisting species, the uptake capacity of different N sources is affected by interspecific competition (e.g., Miller et al., 2007; Simon et al., 2014). Plants have evolved different nutrient acquisition strategies, and the most important one is niche differentiation, which shows that there are preference differences among species for different forms of N to avoid competition (Simon et al., 2017; Huangfu et al., 2019). For example, Huangfu et al. (2019) found that exotic plant *Flaveria bidentis* and coexisting species *Amaranthus retroflexus* preferred to take up N in the form of NO${}_{3}^{-}$ in monoculture, while the former tended to increase the acquisition of NH${}_{4}^{+}$, while the latter had more uptakes of NO${}_{3}^{-}$ when they were grown together in competition. So the flexibility of N acquisition is considered to be an important strategy for plants to reduce niche overlap and mitigate N competition (niche partitioning) to achieve coexistence (McKane et al., 2002; Ashton et al., 2010). However, researches testing for niche separation achieved mixed results (Kuster et al., 2016, references therein), as ^15^N uptake by plants under interspecific competition can be either increased or decreased (Hong et al., 2017).

On the other hand, environmental conditions can cause this inconsistency in plant N acquisition strategy. Plant preference for N is closely related to relative availability of different N forms in soil solution (Zhang et al., 2018). For example, Schimel and Bennett (2004) reported that, with the change of available N supply (such as from boreal forest to tropical forest), the N form preference of plant community switches from organic N to NH${}_{4}^{+}$ and to NO${}_{3}^{-}$. In inorganic N deficient soils, plants tend to use organic N, while in high fertility soils, they use inorganic ones (Scott and Rothstein, 2011). Water content is also related N transformation processes within the soil as it affects the aeration. In aerobic and arid soils, NO${}_{3}^{-}$ is usually the predominant N form available to plants (Xu et al., 2011), whereas NH${}_{4}^{+}$ is the main available N source in flooded and anaerobic soils (such as paddy fields, Houlton et al., 2007). Due to the different transport capacity of different forms of N, water shortage can reduce the N transport to the root surface, thus affecting the uptake process. On the contrary, higher soil water content is conducive to the transformation of organic matter to produce low molecular-weight organic N (e.g., glycine) for plant acquisition (Dijkstra et al., 2015). Consequently, plant uptake of organic or/and inorganic N differs in various ecosystems (Inselsbacher and Näsholm, 2012).

In response to the environmental variation, plants have evolved various root functional traits in term of the N uptake capacity (Hong et al., 2018). Among these, both fine root traits (Legay et al., 2020) and their associated mycorrhizal fungi (Liese et al., 2018) play crucial roles in soil N acquisition, facilitating species coexistence via promoting diversity of resource acquisition strategies (Lambers et al., 2008; Zemunik et al., 2015). In the littoral zone of Shengjin Lake, approximately 17% of the total area consists of *Carex* meadow (Cao and Fox, 2009) which was historically dominated by *Polygonum criopolitanum* one decade ago. As a result, these species coexist in various plant densities. The difference of N acquisition strategy may be affected by the competition regime among species, severing as a potential mechanism driving the community composition. However, we are lacking evidence of whether niche complementarities of inorganic and organic N acquisition exist, and how N acquisition respond to plant competition, as well as the roles of these root functional traits and neighbor density on the outcome of this competition in this wetland ecosystems. In this study, we aimed to test how plant competition influenced the acquisition of different forms of N by two species with different growth strategies, and the possible density dependence involved. We conducted two experiments consisting of a hydroponic test of excised roots of two dominant species (*Carex thunbergii* and *P. criopolitanum*), and an *in situ* experiment in the littoral zone of Shengjin Lake. We proposed the following three hypotheses: (1) the two species had different physiological preferences for N forms in the absence of interspecific competition; (2) root functional traits would contribute to the plant nutrient acquisition with species having more exploitative traits (e.g., high specific root length and root N content) being more efficient to take up N, and *vice versa*; (3) N uptake capacity of focal species was affected by plant competition in a density-dependent and species-specific way.

## Materials and Methods

### Study Site

The Shengjin Lake National Nature Reserve (30° 15′ N-30°30′ N, 116°55′ E-117°15′ E) is located in the southern Anhui Province, China. It is the main habitat for overwintering waterfowls (Meng et al., 2020). The climate in this area belongs to subtropical monsoon. The mean annual precipitation is *ca*. 1600 mm, most falling between May and August, and the mean annual air temperature is 16.4°C. Soils belong to Inceptisols according to the American soil taxonomy (Shi et al., 2004). Multiple shallow ephemeral wetlands were formed due to summer monsoonal flooding and drawdown in water levels during the autumn through spring of the coming year. The littoral zone plants communities mainly consisted of *C. thunbergii, P. criopolitanum, Echinochloa caudata, Miscanthus floridulus, Paspalum distichum, Artemisia annua, Alternanthera philoxeroides*, and *Rumex dentatus* (L.) (Meng et al., 2020). We selected two co-existing plant species that varied in their abundances: *C. thunbergii* and *P. criopolitanum. C. thunbergii* is generally more abundant at higher elevation, and a deep-rooted rhizomatous clonal sedge hygrophyte (Cyberaeae, accounting for more than 70% of total coverage across the littoral zone), and the forb *P. criopolitanum*’s abundance increases with decreasing elevation (accounting for about 5% of total coverage). As such, the vegetation is also known as “*Carex* meadow.” *P. criopolitanum* (Polygonaceae) is mycorrhizal (Wang and Qiu, 2006), while Cyberaeae is primarily non-mycorrhizal (Smith and Read, 2008). In this ecosystem, these two species have a common phenology characterized by two growing periods: the first growing season begins following flood recession in autumn; they spend the winter dormant, then resuming active growth from spring to summer (Yuan et al., 2017). Initial soil physical and chemical properties were listed in Table 1.

**TABLE 1:** Initial soil physical and chemical properties at sampling site of this study.

Total nitrogen (g kg^–1^)	Total phosphorus (g kg^–1^)	pH (H_2_O)	NH${}_{4}^{+}$ (mg kg^–1^)	NO${}_{3}^{-}$ (mg kg^–1^)	Glycine (μ g kg^–1^)
0.22 ± 0.04	0.11 ± 0.008	5.76	4.85 ± 0.50	4.70 ± 0.44	29.7 ± 3.6

### Laboratory Hydroponic Experiment

The usage of excised root segments in hydroponic experiment allowed to characterize the physiological uptake for different N sources in the absence of interspecific competition and to test our hypothesis 1 (Legay et al., 2020). The experiment consisted of culture of excised fine roots of two focal species in vials in a completely randomized factorial design considering two species and four N isotope labeled solutions. We used a total of 40 experimental units with five replicates per treatment (2 species × 4 solutions × 5 replicates) = 40 vials. The four solutions contained three forms of N, NO${}_{3}^{-}$, NH_4_^+^, and glycine in combination (1:1:1) with same total N concentration (500 μM), but only one form of the three N sources was ^15^N labeled in the first three solutions, while the fourth had no ^15^N labeled as a control treatment to account for the natural abundance of ^15^N. This concentration supplied enough N for the test period and allowed us to test for physiological N uptake. Though many organic N sources are shown to be directly absorbed by plants, glycine was repeatedly reported to be abundant amino acid in soils and represent a model organic N source, and recent study has found that glycine is among the organic N with the fastest uptake rate, mainly due to its high mobility (Tian et al., 2020).

Plants were collected from the Shengjin Lake Experimental Station in the fall of 2019. We collected plant-soil monoliths (10 cm width × 10 cm length × 10 cm depth) of target species grew in monoculture in the field using a spade. The monoliths were immediately transported to the laboratory and immersed in water until the soil had been loosely dispersed in the water, and the roots could be easily separated. The intact roots of each species were then washed carefully with tap water. In this study, we sieved and collected only fine roots (<2 mm in diameter), and transplanted to the test vials. In addition, subsamples were stored in refrigerated containers at 4°C for root functional traits analysis (see below). Excised root segments (around 6 cm in length each) of each species were randomly assigned to vials. To minimize the potential decline in N uptake ability, root N uptake of *C. thunbergii* and *P. criopolitanum* started within 30 min after excision. Samples were incubated in one of four solutions with a ^15^N excess of 99% atom. Solution volumes (50 ml) and fresh weights of excised roots (*ca.* 1.5 g in fresh) were selected to maintain a general constant concentration of N over experimental period. We added 0.5 mM CaCl_2_ to maintain membrane integrity. After 2 h, roots were repeatedly washed for 180 s with a 1 mM CaCl_2_ to remove any ^15^N label absorbed on the root surface, followed by rinsing using running demineralized water. Root samples were then oven-dried in paper bags at 65°C for 72 h, ground (MM2, Retsch, Haan, Germany) to a fine powder and aliquots of dried sample (ca. 2 mg) was analyzed by IRMS for ^15^N atom% and N concentrations (Huangfu et al., 2019).

### *In situ* ^15^N Labeling Experiment

To determine whether competition regime modified plant N-form preference (hypothesis 3), the *in situ* ^15^N labeling experiment was conducted at Yang’etou of Shengjin Lake Experimental Station in November 2019, where two species also co-occurred at various abundances in the intermediate elevation in a mosaic pattern, allowing the effect of plant interspecific competition at different densities on N uptake to be tested.

To test whether neighbor density affected ^15^NH${}_{4}^{+}$, ^15^NO${}_{3}^{-}$, and ^15^N-glycine acquisition by plants, we established three blocks of 60 labeling plots (10 cm × 10 cm) in a randomized 4 × 5 factorial design within 0.2 ha area (200 m × 10 m) in the littoral zone. Blocks (10 m × 10 m each) were relatively flat in topography and homogeneous in vegetation composition, and at least 10 m away from the nearest blocks. ^15^N-NO${}_{3}^{-}$, ^15^N-NH${}_{4}^{+}$, and ^15^N-glycine and control treatments were randomly assigned to plots within each block. To reflect field dominance patterns, the plant composition consisted of five intra- and interspecific treatments with various densities of two species, from monodominant to co-dominant communities, each with four individual plants: (*C. thunbergii*: *P. criopolitanum* at 4:0, 3:1, 2:2, 1:3, 0:4). At the time of labeling, the microclimatic conditions and growth stage were similar. We carefully selected target species of similar size and marked them to clarify which treatment was applied. The plant individuals within each plot grew as near as possible to ensure that they could compete for soil resources from each other, while non-target plants were eliminated using scissors (Miller et al., 2007). Surrounding vegetation was clipped to the ground within a 10 cm radius of focal species to limit their interactions in term of N acquisition (Ashton et al., 2010). This was conducted one month prior of N labeling, with repeated clipping to control the regrowth. Tracer solutions were prepared as above, except that each labeling solution consisted of equal concentrations (2 mg N plot^–1^ for each N form) of NH${}_{4}^{+}$, NO${}_{3}^{-}$, and glycine. The amount of N added to the plot was small and minimized the possible N fertilization effect on plant growth as compared to background total N content (6 mg N plot^−1^≈2.7 mg N kg^–1^ soil ≪ 150 mg N kg^–1^ soil), while allowed the detection of ^15^N within plant biomass. A solution equivalent to 6 mg ^15^N was injected into one plot at four equal squares to a depth of 10 cm using a glass syringe, with each square receiving 10 mL of labeled solution, thus adding a total of 40 mL per plot. Higher solution volume was used to increase its diffusion to uniformly label the soil profile. Each injection point was equally spaced from each other within a 2 cm radius of the nearest target plant. Before each injection, we drilled a hole at 10 cm soil depths with a screw-driver to avoid clogging of the needle. Injections were applied on November 7, 2019, when maximal growth was assumed with the mean daily temperature was around 23°C. This date of the experiment was carefully chosen as no rainfall occurred within 5 days before the N treatment to minimize hydrologic transfers of labeled solution within soil column. Considering potential diurnal effects in N uptake, *in situ* labeling was always conducted between 09:00 and 11:00, coinciding with peak photosynthetic activity.

### Plant and Soil Sampling and Analyses

Two hours after ^15^N labeling, leaves and stems were individually harvested using scissors for both species. Thereafter, we collected plant-soil monoliths measuring 10 cm × 10 cm × 15 cm in each plot, and collected the belowground parts (root, rhizome) by washing with deionized water. The sampled plant materials were placed in a portable icebox before transportation to the laboratory within 2 h. In the laboratory, the roots were immediately rinsed with a 1 mM CaCl_2_ to remove any ^15^N label absorbed on the root surface, followed by rinsing with running demineralized water, and finally all plant materials were oven-dried at 60°C for 72 h, ground and analyzed for total N and ^15^N/^14^N ratios as mentioned above.

Fresh fine root subsamples in hydroponic experiment were placed in a transparent tray and covered with deionized water. The tray was scanned using an Epson Expression 10,000 XL photo scanner at 600 dpi (Seiko Epson Corporation, Japan) and analyzed for each species using WinRhizo software (Winrhizo, Regent Instruments, Inc., Québec, QC, Canada) to obtain root length (cm). This information was used to calculate specific root length (SRL; Hong et al., 2018). We also measured the fresh and dry weights of each root sample. Altogether, we obtained root functional traits, including root dry matter content (RDMC, mg/g), SRL (m/g), root ^15^N natural abundance (%) and N content (RNC, mg/g). Among these, RDMC was calculated by dividing dry mass by fresh mass, and SRL was calculated by dividing the length of the roots by their dry mass. We also set aside subsample (*ca*. 0.05 g) of fine root from each species to determined root colonization by AM fungi as we previously described (Huangfu et al., 2019), and root AM fungi colonization (%) was calculated.

We collected bulk soil samples to a depth of 15 cm with five replications before labeling, sieved (2 mm), and stored at 4°C for measurement of soil properties. The characteristics of soils including moisture content (40.92 ± 2.71%) and temperature (28 ± 1.24°C) were measured *in situ* when the labeling was done. Soil total N was analyzed according to the Kjeldahl digestion procedure (Bremner and Tabatabai, 1972). NO${}_{3}^{-}$ and NH${}_{4}^{+}$ was extracted with 2 M KCl and determined using an auto-analyzer (AA3, Bran-Luebbe, Germany). The soil glycine content was also determined in the extracts (Näsholm et al., 1987) using an HPLC (Waters 515, Waters Inc., United States).

### Calculations and Statistical Analysis

The ^15^N atom percent excess (APE) was calculated as the difference of the atom% ^15^N in plants between the ^15^N labeled and control treatments:

(1) $$\begin{array}{c} \text{APE}\left(\%\right)=\text{atom}{\%}_{\text{plant}\left(\mathrm{labeled}\right)}-\text{atom}{\%}_{\text{plant}\left(\mathrm{control}\right)} \end{array}$$

The net uptake rates of N for each plant species (NUR, μg N g^–1^ d.w. root h^–1^) were calculated following McKane et al. (2002) and Miller and Bowman (2003):

(2) $$\begin{array}{c} \text{NUR}=\frac{N\mathrm{content}\left(\mu g/g\right)\times \left(\text{APE}÷100\right)}{\text{time}\left(h\right)\times \left({\text{atom}\%}^{15}\text{N}÷100\right)\times {\text{mass}}_{\text{root}}\left(g\right)} \end{array}$$

The ^15^N uptake (μg ^15^N plant^–1^), defined as the amount of ^15^N recovered from N pool (whole plant), was calculated by multiplying APE (%) with the moles of N in the plants as follows:

(3) Nuptake15=APE×N%plant×Biomassatom%plant(labeled)×15+(100%-atom%plant(labeled))×14×15

The percentage of ^15^N recovered in plants (^15^N_*recovery*_) was calculated using the following equation:

(4) Nrecovery15(%)=Nuptake15Nadded15×100%

where ^15^N_*uptake*_ (μg ^15^N plant^–1^) refers to the ^15^N mass uptake by plants, and ^15^N_*added*_ (μg ^15^N plant^–1^) refers to the total ^15^N mass added to the soil per plot. The contributions of each N form were calculated by dividing the N uptake rate by the sum of uptake rates of the three N forms.

The normal distribution of the data and the homogeneity of variances were tested using the Kolmogorov–Smirnov and Levene’s test, respectively. For the hydroponic experiment, we used the two-way analysis of variance (ANOVA) to estimate the effects of species, N form (the main factors) and their interactions on the N uptake rates. A significant interaction between species and N form would indicate that plant took up different N sources in a species-specific way. To test hypothesis 1, differences between N forms were established according to the Tukey’s HSD *post hoc* test. Data of the two species were analyzed separately. To test hypothesis 2, we also investigated the relationships between root functional traits and total N uptake rate as well as uptake rates of different forms of N using Pearson correlation analysis. The differences in root traits and AM fungi colonization between species were determined using paired *t-*test. For the *in situ* experiment, three-way ANOVA was used to test plant composition, species, N form (the main factors) and their interactions on N uptake rates and ^15^N recovery separately, followed by the Tukey *post hoc* test. An interaction between plant composition and N form would indicate different N acquisition strategies depending on competition regimes. To test hypothesis 3, the difference in contribution of each N form (calculated by dividing the N uptake rate by the sum of uptake rates of the three forms) among different plant combinations were determined by one-way ANOVA for each species. Differences in total biomass and the root to shoot ratios for the two species were analyzed by two-way ANOVA, testing the main and interactive effects of species and competition regime. We performed all statistics using the SPSS software 17.0 (IBM Inc., Chicago, IL, United States). Figures were designed using OriginPro 9.1. Differences were tested for significance at *P* = 0.05.

## Results

### The Root Functional Traits

All root functional traits except ^15^N natural abundance (measured in subsection of “Plant and Soil Sampling and Analyses”) were significantly different between the two plant species. *C. thunbergii* had a significantly higher mean RDMC (210 vs 40 mg/g), lower SRL (32.10 vs 51.75 m/g), lower N concentration (6.43 vs 16.45 mg/g), and AM fungi colonization than *P. criopolitanum* (4.39 vs 73.5%, Table 2).

**TABLE 2:** Variation of root functional traits of two plant species (Mean ± SE) tested by paired *t*-test. RNC, SRL, and RDMC indicated root nitrogen content, specific root length, root dry matter content, and AM fungi colonization, respectively.

Root traits	*Carex thunbergii*	*Polygonum criopolitanum*	*P*
^15^N natural abundance (%)	0.36 ± 0.002	0.38 ± 0.006	0.435
RNC (mg/g)	6.43 ± 0.52	16.45 ± 1.41	0.004
SRL(m/g)	32.10 ± 1.82	51.75 ± 2.13	0.005
RDMC(mg/g)	210 ± 15.14	40 ± 5.52	0.001
AM fungi colonization (%)	4.39 ± 0.16	73.5 ± 6.84	0.001

### The Plant Uptake Rates for Different N Forms: Hydroponic Experiment

**FIGURE 1:**
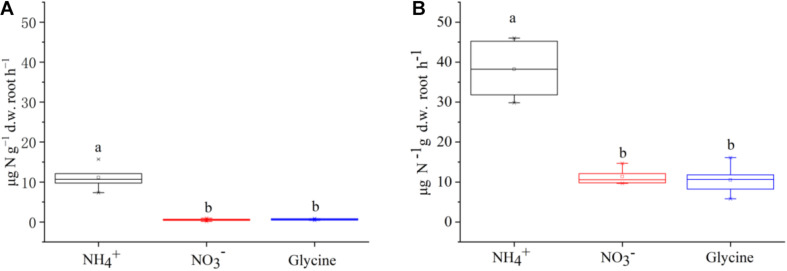
Uptake rates of glycine, NO${}_{3}^{-}$, and NH${}_{4}^{+}$ by *Carex thunbergii* **(A)** and *Polygonum criopolitanum* **(B)** using excised roots under different treatments. Values are presented as means ± SE of five replicates. Different lowercase letters indicate significant differences in uptake rates among N forms for each species (one-way ANOVA, *P* < 0.05).

The net N uptake rates (including NH${}_{4}^{+}$, NO${}_{3}^{-}$, and glycine-N) differed among two plant species (*F* = 135.13, *P* < 0.001) and N forms (*F* = 84.42, *P* < 0.001). The sum of net uptake rates of three N forms was almost four times higher for *P. criopolitanum* than for *C. thunbergii* (60.12 vs 12.30 μg N g^–1^ d.w. root h^–1^, Figure 1). Further, there were significant interactions of plant species × N form (*F* = 16.63, *P* < 0.001). To be specific, both species took up N in the form of NH${}_{4}^{+}$ at the highest rate (*P* < 0.05). While NO${}_{3}^{-}$ and glycine-N contributed over one third to net N uptake for *P. criopolitanum*, they only contributed around 9% to net N uptake for *C. thunbergii*.

#### The Effect of Plant Competition on Plant Growth and Uptake Rates of Different N Forms: *in situ* ^15^N Labeling Experiment

**FIGURE 2:**
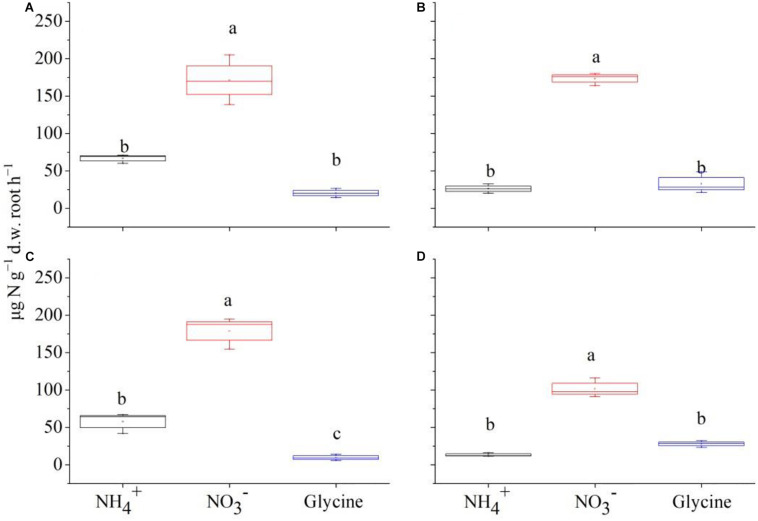
The mass-specific net rate of nitrogen (μg N g^–1^ d.w. root h^–1^) in the form of NH${}_{4}^{+}$, NO${}_{3}^{-}$, and glycine-N by *Carex thunbergii* under competition regime of 4:0 (*C. thunbergii*: *Polygonum criopolitanum*, **A**), 3:1 **(B)**, 2:2 **(C)**, and 1:3 **(D)**, respectively. The values are means ± SE of three replicates. Different letters above each symbol indicate significant differences of mass-specific net rate of N uptake among three different N forms within a specific competition regime treatment at *P* < 0.05.

**FIGURE 3:**
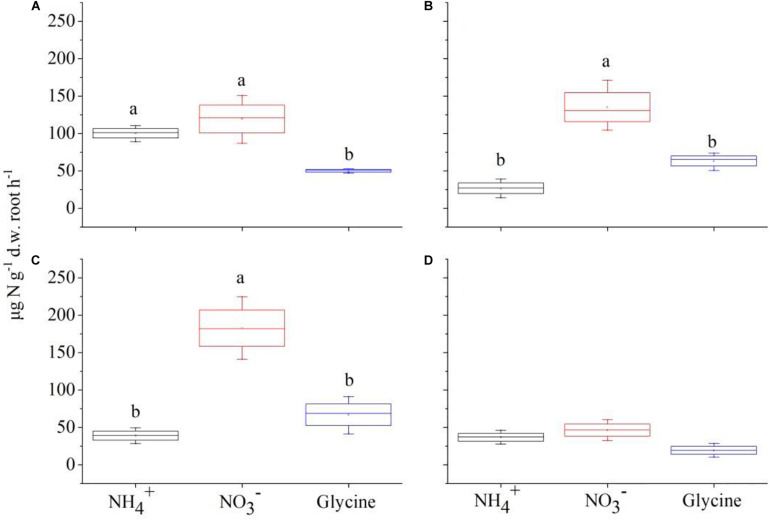
The mass-specific net rate of nitrogen (μg N g^–1^ d.w. root h^–1^) in the form of NH${}_{4}^{+}$, NO${}_{3}^{-}$, and glycine-N by *Polygonum criopolitanum* under competition regime of 4:0 (*Polygonum criopolitanum*: *Carex thunbergii*, **A**), 3:1 **(B)**, 2:2 **(C)**, and 1:3 **(D)**, respectively. The values are means ± SE of three replicates. Different letters above each symbol indicate significant differences of mass-specific net rate of N uptake among three different N forms within a specific competition regime treatment at *P* < 0.05.

There were no significant difference among plant combinations in total biomass for either species (*F* = 1.57, *P* = 0.205). However, interspecific competition altered their biomass allocation patterns. Specially, *P. criopolitanum* allocated more biomass into belowground part with increasing density of competitor, leading to higher root to shoot ratio (Supplementary Figure 1, *P* < 0.05). Net uptake rates of different N forms were significantly affected by species, plant composition, N form, and their interactions (Table 3, all *P* < 0.05 except for species × plant composition). With the exception of combinations dominated by either species, net uptake rates of inorganic N were comparable for *C. thunbergii* and *P. criopolitanum*, and higher for NO${}_{3}^{-}$ than for NH${}_{4}^{+}$. The comparisons of uptake rates for glycine were largely species- or plant composition-dependent (Figures 2, 3).

**TABLE 3:** The three-way analysis of variance (ANOVA) results for the nitrogen (N) uptake rates of *Carex thunbergii* and *Polygonum criopolitanum* with plant composition (C), species (S), and N form (F) as the main factors.

Source of variance	df	*F*	*P*
Plant composition	3	28.09	<0.001
N form	2	235.86	<0.001
Species	1	3.94	0.014
C × F	6	12.90	<0.001
S × C	3	1.83	0.154
S × F	2	19.22	<0.001
S × C × F	6	3.75	0.004

For *C. thunbergii*, in contrast to monoculture, interspecific competition significantly decreased NH${}_{4}^{+}$ uptake rates at plant combination of 3:1 and 2:2 (*C. thunbergii*: *P. criopolitanum*), while the uptake rates of NO${}_{3}^{-}$ and glycine were not affected (Figures 2B,C). However, when the community was dominated by *P. criopolitanum* (at 1:3), the uptake rates of both NO${}_{3}^{-}$ and NH${}_{4}^{+}$ were greatly reduced (*P* < 0.05, Figure 2D). Relatively, *P. criopolitanum* in monoculture acquired N in the form of NO${}_{3}^{-}$ and NH${}_{4}^{+}$ at comparable rates. Similarly, interspecific competition from *C. thunbergii* significantly decreased NH${}_{4}^{+}$ uptake rates at plant composition of 3:1 and 2:2 (*P* < 0.05, Figures 3B,C). Finally, interspecific competition significantly decreased N uptake rates in the form of NO${}_{3}^{-}$ by *P. criopolitanum* when two species growing at 3:1, as a result, three forms of N contributed equally to the total N uptake (*P* > 0.05, Figure 3D).

### Recovery of ^15^N by Plants

**FIGURE 4:**
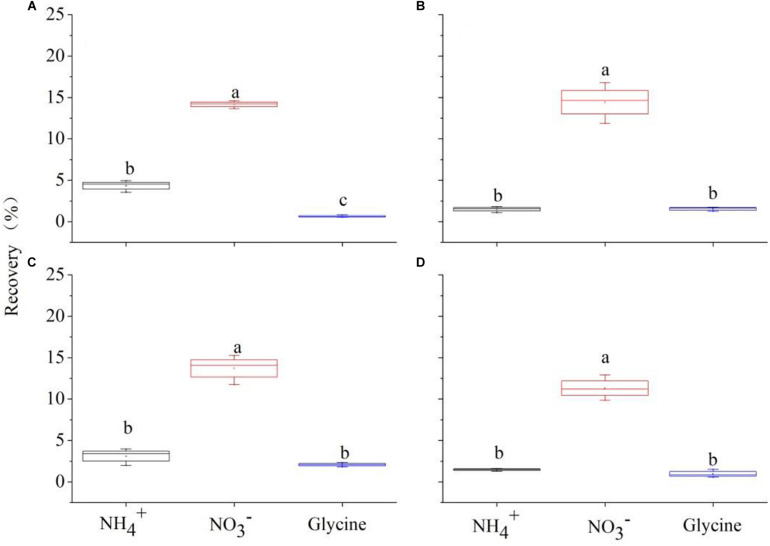
^15^N recovered by *Carex thunbergii* (% of added ^15^N) from NH${}_{4}^{+}$, NO${}_{3}^{-}$, and glycine-N 2 h after ^15^N injection under competition regime of 4:0 (*Carex thunbergii: Polygonum criopolitanum*, **A**), 3:1 **(B)**, 2:2 **(C)**, and 1:3 **(D)**, respectively. The bars and error bars show means ± SE (*n* = 3).

**FIGURE 5:**
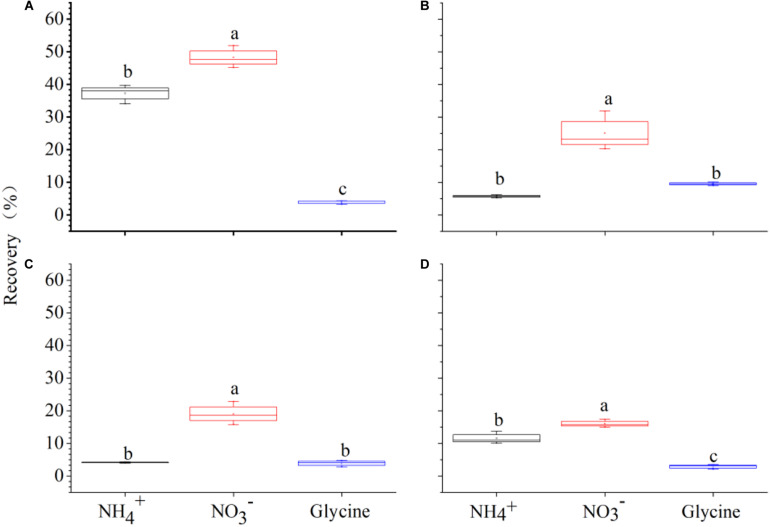
^15^N recovered by *Polygonum criopolitanum* (% of added ^15^N) from NH${}_{4}^{+}$, NO${}_{3}^{-}$, and glycine-N 2 h after ^15^N injection under competition regime of 4:0 (*Polygonum criopolitanum*: *Carex thunbergii*, **A**), 3:1 **(B)**, 2:2 **(C)**, and 1:3 **(D)**, respectively. The bars and error bars show means ± SE (*n* = 3).

Two-way ANOVA showed that species (*F* = 402.58, *P* < 0.001) and plant composition (*F* = 104.98, *P* < 0.001), as well as their interaction (*F* = 89.09, *P* < 0.001) had significant effects on total N recovery (%). Specifically, *P. criopolitanum* generally recovered more ^15^N than *C. thunbergii*. In the monoculture treatment, for example, total ^15^N recovery in *P. criopolitanum* (89.53 ± 3.27%) was four times higher than in *C. thunbergii* (19.12 ± 0.56%) 2 h after labeling (Figures 4, 5, *P* < 0.05). Overall, plant composition did not affect total ^15^N recovery by *C. thunbergii* with the exception when growing at 1:3 (13.73 ± 0.54%, *F* = 6.81, *P* = 0.014, Figure 4D). On the contrary, the total ^15^N recovery in *P. criopolitanum* decreased with the increase of competitor density (40.34 ± 3.39%, 27.17 ± 2.06%, 30.55 ± 2.18% for 1:3, 2:2, 3:1, respectively, *F* = 107.52, *P* < 0.001, Figure 5).

Relatively, N form had a more important effect on ^15^N recovery (*F* = 482.62) than species (*F* = 463.47) and plant composition (*F* = 120.86), and ^15^N recovery was also influenced by its interactions with species and plant composition (all *P* < 0.001, Table 4). For *C. thunbergii*, its N recovery under competition was context-dependent. When growing with low and medium competition from *P. criopolitanum* (at 3:1 and 2:2 ratios), *C. thunbergii* increased N recovery in the form of NH${}_{4}^{+}$ and glycine while recovery of NO${}_{3}^{-}$ was generally stable. When the competition continued to increase (i.e., at 1:3 ratio), both NH${}_{4}^{+}$ and NO${}_{3}^{-}$ were reduced (Figure 4), leading to an overall decrease in total N recovery. In contrast, interspecific competition had a remarkable effect on ^15^N recovery in any form by *P. criopolitanum* (*P* < 0.05, Figure 5). Consequently, ^15^N recoveries of NH${}_{4}^{+}$ and NO${}_{3}^{-}$ by *P. criopolitanum* were greatly reduced under competition compared to the monoculture. The recovery of NO${}_{3}^{-}$-^15^N was reduced by 48.06% (at ratio of 1:3) to 66.74% (at ratio of 3:1, all *P* < 0.05). Collectively, the uptake of glycine by *P. criopolitanum* was significantly enhanced in the presence of *C. thunbergii* (Figure 5B, *P* < 0.05). Similar results were observed for NH${}_{4}^{+}$ at ratio of 3:1, leading to overall similar total N recovery among three competition treatments (Figure 5, *P* > 0.05).

**TABLE 4:** The analysis of variance (ANOVA) results for the ^15^N recovery of *Carex thunbergii* and *Polygonum criopolitanum* with plant composition (C), species (S), and N form (F) as the main factors.

Source of variance	df	*F*	*P*
Plant composition	3	120.86	<0.001
N form	2	482.62	<0.001
Species	1	463.47	<0.001
C × F	6	42.56	<0.001
S × C	3	102.56	<0.001
S × F	2	45.26	<0.001
S × C × F	6	29.62	<0.001

**FIGURE 6:**
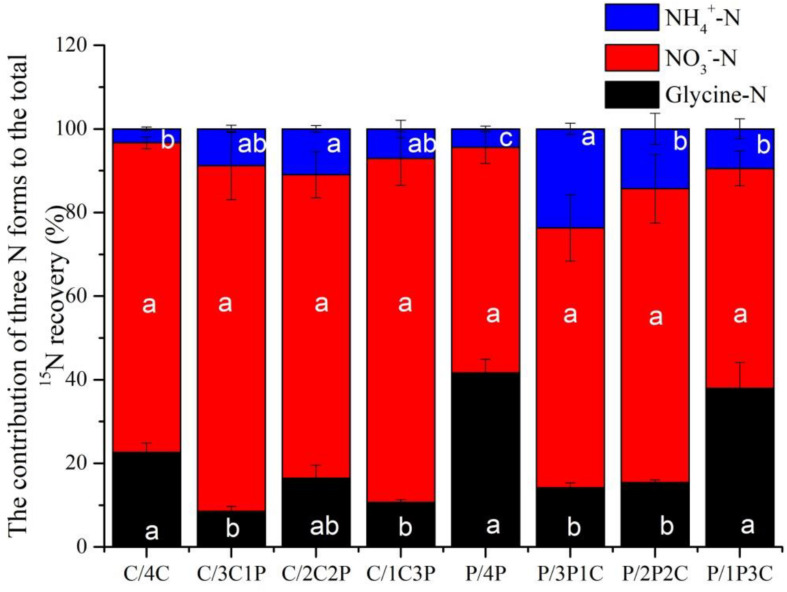
Contributions of N recovery in three forms for *Carex thunbergii* and *Polygonum criopolitanum* in the field experiment. Treatment codes: C and P signify *C. thunbergii*, and *P. criopolitanum* individuals, respectively, followed by the specific combination tested, in which the composition of each component species is indicated by numerals. Values indicate the mean ± SE (*n* = 3). For each species, bars with different lowercase letters denote a significant difference between combinations within same N form at *P* < 0.05.

Although competition did not change the order of uptake preference for the three N forms among two focal species, they did change the contributions of three N forms (Figure 6). For all treatments, uptake of NO${}_{3}^{-}$ accounted for more than 50% of total N uptake (sum of the uptake of the three N forms), with the highest value for *C. thunbergii* (77.91% on average). The difference of two species in N acquisition observed was mainly associated with the contributions of NH${}_{4}^{+}$ and glycine. Though competition increased the uptake of glycine, its contribution to N recovery by *P. criopolitanum* seemed to be more important (Figure 6, *P* < 0.05). The contribution of glycine to *P. criopolitanum* was increased by 19.28, 9.92, and 5.06% of the total N uptake at ratios of 3:1, 2:2, 1:3, respectively, and was significantly higher than that to *C. thunbergii* (5.47, 7.65, and 3.72%, Figure 6, *P* < 0.05). While two plant species decreased NH${}_{4}^{+}$ uptake under competition, the contribution of NH${}_{4}^{+}$ to *P. criopolitanum* was nearly 38% of the total N uptake, which was comparable to the control with no competition (41%, Figure 6, *P* > 0.05).

### Correlations Between Root Traits and N Uptake Rates (Hypothesis 2)

Correlation analysis revealed that both RNC and SRL were significantly positively correlated with the total N uptake rate and its components (all *r* > 0.75, *P* < 0.01), and RDMC was significantly negatively correlated with these variables (*r* < −0.85, *P* < 0.01). There was no significant relationship between the ^15^N natural abundance value and N uptake rates (*P* > 0.50, Supplementary Table 1).

## Discussion

Our results provided evidence of niche complementarity for the resource use of species regulated by competition regimes. Two species differed in their N acquisition strategies in response to competition: both preferred NO${}_{3}^{-}$ when grown in monoculture, while in the presence of competitors, the preference of fast-growing *C. thunbergii* persisted but *P. criopolitanum* acquired more NH${}_{4}^{+}$ and glycine-N, especially with increasing neighbor densities.

### N Uptake Variations Dominated by N Form

Previous researchers revealed that N uptake is related to N form, N availability in soils, and species identity (Britto and Kronzucker, 2013). In our *in situ* study, N form acted as the dominant factor driving N uptake, and plant species showed various influences. Though it was often found that plants obtain N from soil proportionally to its availability, contrary to our hypothesis one, both species in this study preferred N in the form of NO${}_{3}^{-}$, followed by NH${}_{4}^{+}$, and lastly glycine, regardless of N availability in the soil. Similar result has been reported with *Fagus grandifolia*, which takes up approximately five times more NO${}_{3}^{-}$ than NH${}_{4}^{+}$, despite the fact that NO${}_{3}^{-}$ content is lower than NH${}_{4}^{+}$ in soils (Templer and Dawson, 2004). Limited niche partitioning of N uptake among the coexisting species has been reported in subtropical ecosystems (Yang et al., 2018), which might be largely explained by species-specific differences (Miller and Bowman, 2003) rather than the relative dominance among N forms in soils (Schimel and Bennett, 2004). Relatively, NO${}_{3}^{-}$ is easily diffused to the root surface through water flow due to the difference in the mobility of different N forms (Hodge et al., 2000), whereas NH${}_{4}^{+}$ is preferably immobilized by microorganisms in the field (Romero et al., 2015). This process is particularly of importance in wetland ecosystems where soil moisture is often higher than that of most terrestrial ecosystems. In this context, sufficient water can selectively enhance the delivery of NO${}_{3}^{-}$ to the root surface, and consequently affect their uptake by plants (Boudsocq et al., 2012).

Another consideration for NO${}_{3}^{-}$ domination is associated with pH of growth media. Plant growth preference for N forms can shift from NO${}_{3}^{-}$ at pH of 5.5 to NH${}_{4}^{+}$ at pH of 6.4 (Teyker and Hobbs, 1992). Therefore, in contrast to NO${}_{3}^{-}$, NH${}_{4}^{+}$ seemed less important, at least for *C. thunbergii* in this system. Compared to extreme ecosystems such as alpine or arctic region, the general less contribution of glycine in the wetland ecosystems might reflect the lower availability of glycine in the soil, largely conforming with habitat filtering selection patterns (Schimann et al., 2008). Our judicious estimates of contribution of glycine to the total N uptakes ranged between 3 and 25% by wetland species was lower than that reported by the dominant species in alpine meadows (13–35%, Xu et al., 2006) and alpine wetlands (20–40%) (Gao et al., 2014).

### Species-Specific N Uptake in Response to Plant Competition

In line with our hypothesis 3, interactions between two species indicated asymmetric competition in term of N uptake pattern: the presence of competitor caused less plasticity in dominant species (*C. thunbergii*) than subdominant species (*P. criopolitanum*), supporting niche preemption hypothesis which predicated that the species with low abundance switches to a different N sources while species with high abundance takes up N mostly from NO${}_{3}^{-}$ (Ashton et al., 2010). Functional traits have been recognized to be related to plant N acquisition strategies (Hong et al., 2018). In this study, the lack of niche partitioning of N acquisition between two species might be explained by their root N niche separation in space instead of chemical form of N (von Felten et al., 2012). *C. thunbergii*, a rhizomatous clonal sedge characterized by high deep rooting and branching frequency (Gao et al., 2012), is capable of exploiting more soil volumes and thus reducing the diffusion path of N to root surfaces (Fitter, 1991). Critically, rhizomes of *C. thunbergii* might be beneficial in assisting N translocation among different clone parts rooting under different nutrient patches (Stuefer, 1998), showing a much stable N acquisition strategy. *P. criopolitanum* is a shallow-rooted forb, which might take up N concentratedly in the upper soil and must shift its N niche when growing with competitors by acquisition of a greater variety of N forms (Mommer et al., 2011), showing a relatively flexible N acquisition strategy. Future work is needed to develop occupancy models for species root systems in natural communities with more root functional traits included.

In spite of this, thinner roots of *P. criopolitanum* (as shown by high SRL) allow it to maximize N capture, leading to more efficient N acquisition (Hong et al., 2018), which was evident in the hydroponic experiment when equal root mass used. In supporting of our hypothesis 2, therefore, root functional traits such as high SRL can be similarly functional or more effective in nutrient foraging than placing roots selectively in nutrient hotspots (Mommer et al., 2011). Considering larger size of *C. thunbergii* than that of *P. criopolitanum*, this seeming counter-intuitive pattern might be ascribed to the luxury N utilization of *P. criopolitanum*, whereby excess N resources were taken up relative to the immediate growth rate (e.g., Liu et al., 2020). The increasing root to shoot ratios of *P. criopolitanum*, along with its higher N content than *C. thunbergii* (Table 2; Meng et al., 2020), provided additional evidence that this forb may have more efficient N uptake ability due to relative exploitative traits.

N niche partitioning could also be mediated by mycorrhizal symbiosis with plant species (Gerz et al., 2018). Previous studies have shown that symbiosis with arbuscular mycorrhizal (AM) fungi which are highly effective at acquiring N, especially in the form of NH${}_{4}^{+}$ (Jin et al., 2005). As such, more recovery of NH${}_{4}^{+}$ by *P. criopolitanum* was likely due to the possibility that the effects of interspecific competition were mitigated by its symbiotic AM fungi in N acquisition and reduced dependency on external N supply. Moreover, increasing glycine uptake by *P. criopolitanum* may be due to the fact that it was subordinate in the soil N pool, as a compensation, help host plant buffer competition from the competitors under N-limited conditions (McKane et al., 2002; Silvertown, 2004; Miller et al., 2007).

### Variations of N Uptake Ability Regulated by the Neighbor Density

One way to test plant species partitioning in resource use is manipulating the neighbor density and evaluate if this manipulation changes the use patterns of interspecific competitor at community level (Silvertown, 2004). In support of our hypothesis 3, the neighbor density significantly influenced N uptake pattern of plant species (thereby N preference), in particular for *P. criopolitanum*. Such a change in N acquisition as a result of the shifts in competition regime has been invoked, but has rarely been tested (Miller et al., 2007). In a recent study, Huangfu and Li (2019) revealed that the density of an invasive species in a community was crucial in determining not only its direct ecological impacts (e.g., growth performance) but also the strength of trophic cascades (e.g., N uptake pattern) of neighboring native species. The density-dependent model predicated that plant growth, as well as associated ecological functions (e.g., nutrient uptake) would decreases as density increases when resource competition is the dominating factor in plant interactions (Barto and Cipollini, 2009). This is the case for the relationship between *C. thunbergii* and *P. criopolitanum* in our study, likely due to their N niche overlap (Huangfu and Li, 2019). Therefore, the finding of lower total N recovery by *C. thunbergii* in competition when *P. criopolitanum* dominated a community compared to monoculture signified a mediation of plant competition. Particularly, slightly higher total N uptake by *P. criopolitanum* at ratio of 3:1 than that at 2:2 could be found when interspecific competition from both species was lowered, assumedly due to the niche shift under competition (Miller et al., 2007). In this case, for instance, the increased planting density of *C. thunbergii* reduced the soil volume for root of *P. criopolitanum*, thereby the selection for separations of N in chemical forms should become strong. Overall, our results indicated there was stronger competition between species for NO${}_{3}^{-}$ than other N forms with increasing neighbor densities, and subdominant species could shift its preference for NO${}_{3}^{-}$ to meet their N demand by flexible uptake for alternative N forms. Recognition of the density-dependency in the provision of essential ecosystem services leads to a new conceptual insight into how competition regime change key ecosystem processes. Furthermore, given that NO${}_{3}^{-}$ was in high demand by focal species, especially *C. thunbergii*, not much of NO${}_{3}^{-}$ would lose via denitrification or leaching at sites where they dominated.

We recognize that only two plant species were included in this study. Consequently, the lack of replication at the species level limited the scope of inference of the results and interpretation of N foraging differences between species. Further research incorporating a large number of wetland species from different functional groups is needed to better understand the relationship between plant functional traits and associated resources acquisition strategies.

## Conclusion and Implications

Two plant species shared common preferences for N sources when grew in monoculture, but they showed divergent responses and species-specific patterns in N acquisition strategies under different competition regimes. Collectively, the higher NH${}_{4}^{+}$ and glycine but lower NO${}_{3}^{-}$ acquisition by *P. criopolitanum* compared to *C. thunbergii* in competition suggested that the subdominant species could have a relative advantage over dominant species via better exploitation of less accessible soil source (Xi et al., 2017). These divergences in N acquisition between two species might be partially explained by different root functional traits and the degree of N uptake plasticity. Therefore, the species coexistence is likely realized under the field conditions via niche partitioning in N uptake (Kahmen et al., 2006).

## Data Availability Statement

The original contributions presented in the study are included in the article/Supplementary Material, further inquiries can be directed to the corresponding author.

## Author Contributions

XJ: methodology, software, validation, formal analysis, investigation, data curation, and writing–original draft. CH: conceptualization, methodology, validation, formal analysis, investigation, data curation, writing, review and editing, and supervision funding acquisition. DH: methodology, validation, and review and editing. All authors contributed to the article and approved the submitted version.

## Conflict of Interest

The authors declare that the research was conducted in the absence of any commercial or financial relationships that could be construed as a potential conflict of interest.
