# Supplementary material for: Nitrogen Uptake by Two Plants in Response to Plant Competition as Regulated by Neighbor Density
Source: Front Plant Sci. 2020 Dec 10;11:584370. doi: 10.3389/fpls.2020.584370 (PMC7758497; doi:10.3389/fpls.2020.584370)
Supplement: Supplementary file 1 [file Data_Sheet_1.docx]

**Supplemental files**

|  | Root nitrogen content (RNC) | Specific root length (SRL) | Root dry matter content (RDMC) | ^15^N natural abundance |
| --- | --- | --- | --- | --- |
| The total N uptake rate | 0.853** | 0.911** | -0.945** | 0.16 |
| N uptake rate in the form of NH_4_^+^ | 0.779** | 0.892** | -0.910** | 0.231 |
| N uptake rate in the form of NO_3_^−^ | 0.914** | 0.930** | -0.950** | 0.142 |
| N uptake rate in the form of glycine | 0.849** | 0.794** | -0.881** | -0.032 |

**Table S1** Pearson Correlation coefficients and significance (** indicates *P* < 0.01; *n* = 5) between root functional traits and N uptake rate variables, measured in the hydroponic test


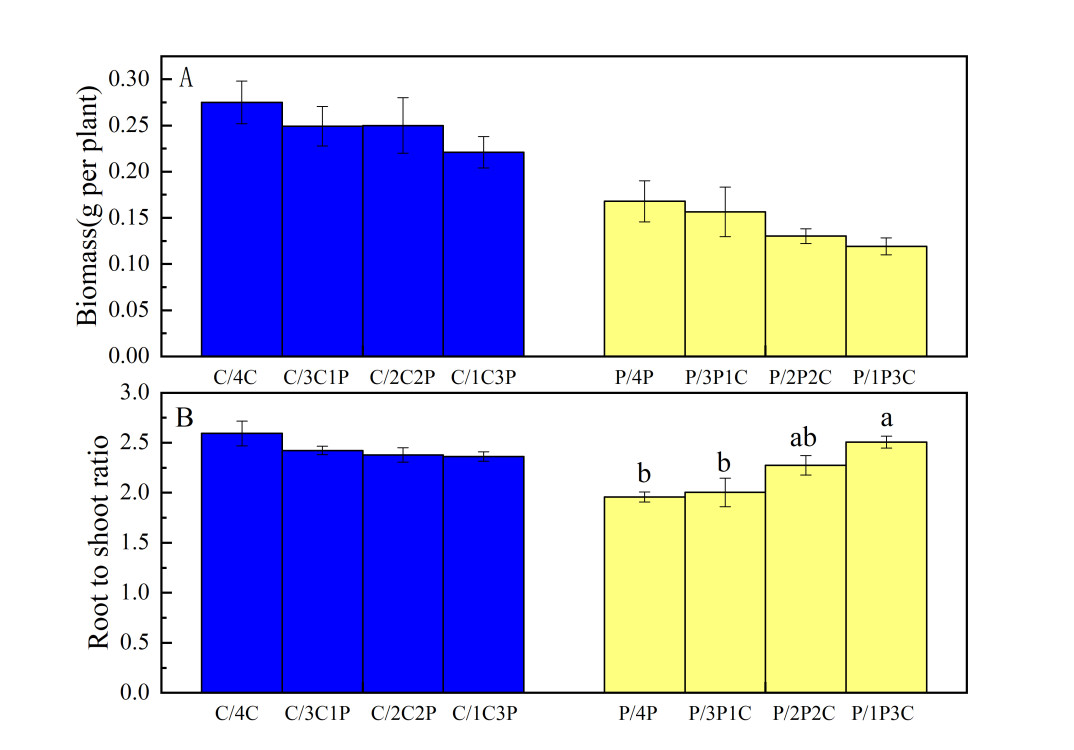


**Fig. S1.** Total biomass (A) and root to shoot ratio (B) of *Carex thunbergii* and *Polygonum cripolitanum* in monocultures and mixtures (mean ± SE). Treatment codes: C and P signify *C. thunbergii*, and *P. criopolitanum* individuals, respectively, followed by the specific combination tested, in which the composition of each component species is indicated by numerals. Bars with different lowercase letters indicate significant differences among the two species and the two cropping regimes at *p* < 0.05 for either species from various plant compositions (*P*< 0.05).
